# Quantifying Adaptive and Innate Immune Responses in HIV-Infected Participants Using a Novel High Throughput Assay

**DOI:** 10.1371/journal.pone.0166549

**Published:** 2016-12-09

**Authors:** Michelle K. Yong, Paul U. Cameron, Tim Spelman, Julian H. Elliott, Christopher K. Fairley, Jeffrey Boyle, Misato Miyamasu, Sharon R. Lewin

**Affiliations:** 1 The Peter Doherty Institute for Infection and Immunity, The University of Melbourne, Melbourne, Australia; 2 Department of Infectious Diseases, Monash University and Alfred Hospital, Melbourne, Australia; 3 Centre for Population Health, Burnet Institute, Melbourne, Australia; 4 Central Clinical School, Monash University and Melbourne Sexual Health Centre, Melbourne, Australia; 5 QIAGEN, Germantown, Maryland, United States of America; University of Pittsburgh Centre for Vaccine Research, UNITED STATES

## Abstract

**Objectives:**

HIV infection is characterised by persistent immune dysfunction of both the adaptive and innate immune responses. The aim of this study was to evaluate these responses using a novel high throughput assay in healthy controls and HIV-infected individuals prior to and following anti-retroviral treatment (ART).

**Design:**

Cross-sectional study.

**Methods:**

Whole blood was assessed using the QuantiFERON Monitor^®^ (QFM) assay containing adaptive and innate immunostimulants. Interferon (IFN)-γ levels (IU/mL) were measured by enzyme-linked immunosorbent assay (ELISA).

**Results:**

We recruited HIV-infected participants (n = 20 off ART and viremic; n = 59 on suppressive ART) and HIV-uninfected controls (n = 229). Median IFN-γ production was significantly higher in HIV-infected participants compared to controls (IFN-γ 512 vs 223 IU/ml, p<0.0001), but within the HIV-infected participants there was no difference between those on or off ART (median IFN-γ 512 vs 593 IU/ml p = 0.94). Amongst the HIV-infected participants, IFN-γ production was higher in individuals with CD4 count>350 compared to <350 cells/μL (IFN-γ IU/ml 561 vs 259 p = 0.02) and in males compared to females (IFN-γ 542 vs 77 IU/ml p = 0.04). There were no associations between IFN-γ production and age, plasma HIV RNA, nadir CD4 count or duration of HIV infection. Using a multivariable analysis, neither CD4 nor sex were independently predictive of IFN-γ production.

**Conclusion:**

Using a high throughput assay which assesses both adaptive and innate immune function, we showed elevated IFN-γ production in HIV-infected patients both on and off ART. Further research is warranted to determine if changes in QuantiFERON Monitor^®^ are associated with clinical outcomes.

## Introduction

Despite significant advances in anti-retroviral therapy (ART), people living with HIV infection continue to have persistent immunological deficits [1]. Although HIV can be rapidly controlled with a recovery of CD4+ T cells to normal level, immune function often does not return to normal with ongoing persistent inflammation and high levels of generalised immune activation in both CD4 and CD8 T cells [2]. These changes are more marked in individuals with lower nadir CD4 T-cell counts [3]. Persistent immune activation or dysfunction is also thought to contribute to long term morbidity and mortality in patients receiving ART [4,5], poor CD4+ T cell recovery and serious non-AIDS events (SNAEs) such as accelerated atherosclerosis and impaired bone mineral density [2,6,7].

A simple standardized assay measuring immune function could potentially be clinically useful to monitor for individuals at high risk of SNAEs, particularly in the setting of a normal CD4+ T-cell count. We evaluated a recently developed high throughput assay which quantifies innate and adaptive cell-mediated immune function (QuantiFERON Monitor^®^, QIAGEN, Germantown, MD, USA). This simple, rapid whole blood assay measures interferon gamma (IFN-γ) production after stimulation with anti-CD3, a CD3 T cell receptor ligand (adaptive) and R848, a TLR7/8 ligand (innate). Recently the QFM assay was evaluated and assessed in a cohort of liver transplant recipients [8] and is currently intended to use for detecting cell mediated immune responses in the immunosuppressed solid organ transplant population.

The aim of this study was to evaluate this assay in HIV-infected and non-infected populations with the hypothesis that elevated immune activation seen in HIV infection would be reflected in this assay of immune function and would persist on suppressive ART.

## Methods

### Study participants

A cross sectional study was performed in HIV-infected participants recruited from The Alfred Hospital and the Melbourne Sexual Health Centre (MSHC), Melbourne Australia. HIV-infected participants off treatment were either ART naïve or off ART for at least 12 months prior to enrolment. HIV-infected participants on treatment were receiving ART for at least 12 months and had two measurements of plasma HIV RNA less than 50 copies/ml in the six months prior to enrolment. The study was approved by the Alfred Hospital Research and Ethics Committee and all patients provided written informed consent. Uninfected controls were recruited by Clinical Trials Connect P/L and did not have any specified medical conditions nor were taking any medications. Study blood was taken at the Skin & Cancer Foundation Victoria with institutional review board approval.

### QuantiFERON Monitor (QFM) ^®^

Whole blood was assessed using the QuantiFERON Monitor^®^ (QFM) assay according to the manufacturer’s instructions. One ml of whole blood was drawn into QuantiFERON blood collection tubes with QFM LyoSpheres containing an anti-CD3 T cell receptor ligand and R848, a TLR7/8 ligand. QFM LyoSpheres were tested in two different concentrations: neat and 1/10 (QFM 1:10). Tubes were then incubated for 16 to 24 hours at 37°C without humidification and the plasma was harvested after centrifugation. Plasma IFN-γ (IU/mL) was measured using an ELISA technique and results calculated using analysis software provided by the manufacturer. The maximum possible response of IFN-γ was 2000 IU/ml and responses greater than this level were not determined.

### Statistical analysis

The primary endpoint was IFN-γ response (IU/ml) to innate and adaptive stimulants (anti CD3 and R848) in HIV-infected relative to HIV-uninfected controls. Secondary analysis included pairwise comparisons of IFN-γ response between HIV-infected participants on and off ART. A formal skewness/Kurtosis test for normality was performed. Univariate analysis were performed using Wilcoxon rank sum tests for non-parametric continuous data. Proportions were compared using a chi square test or Fisher’s exact test as appropriate. Multivariate analysis was performed using a quantile median regression to investigate correlates. Candidate predictors for inclusion into the multivariate model included significant outcomes on univariate analysis and clinical factors which the literature suggests may impact test outcome. A p-value <0.05 was considered to be statistically significant. All analyses were performed using GraphPad Prism software (v6; San Diego, California, USA) and Stata Version 12.0 (Statacorp, College Station, Texas, USA).

## Results

### Participant characteristics

Seventy-nine HIV-infected study participants (n = 20 off ART and n = 59 on ART) and 229 HIV-uninfected controls were enrolled. The median age of HIV-infected participants was significantly older than controls (45 [IQR 34–55] vs 30 [25–44.5] years, p<0.0001). There were also more female participants in the control group compared to the HIV-infected (57.9% vs 5.1% respectively, p<0.0001). HIV-infected participants on suppressive ART were significantly older than HIV-infected participants off ART (47 [IQR 41–56] vs 31 [IQR 26–41] years, p<0.001) and the participants on ART correspondingly had a longer duration of known HIV infection (median 12.3 vs 1.4 year, p = 0.004). The median CD4+ T-cell count at the time of study enrolment was 600 cells/μl in participants on ART compared to 518 cells/μl in participants off ART (p = 0.18). The median plasma HIV RNA of participants off ART was 25,903 copies/ml compared to <50 copies/ml in participants on ART (p<0.001) and participants on ART had a median nadir CD4+ T-cell count of 210 cells/μl. Median duration on ART was 11.4 years.

### IFN-γ production in HIV infection

**Fig 1:**
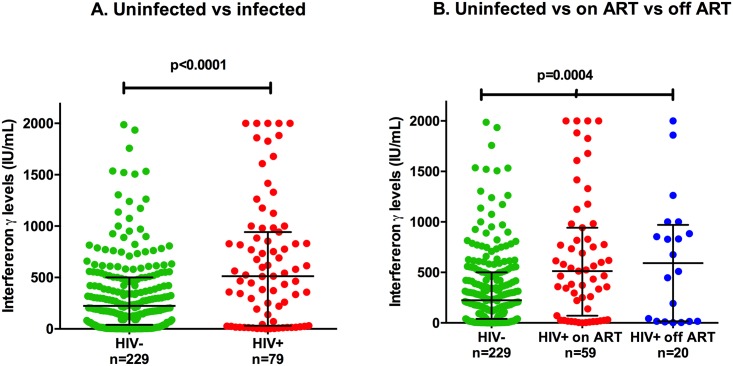
Comparison of innate and adaptive IFN-γ production ex vivo with horizontal error bars representing the median and interquartile range. A. HIV-uninfected controls and HIV infected participants B. HIV-uninfected controls and HIV-infected participants on and off ART.

Using the QuantiFERON LyoSpher 1:10 (QFM 1:10) assay, IFN-γ production was significantly different in the three populations studied (median IFN-γ on ART vs off ART vs HIV-uninfected 512 vs 593 vs 223 IU/ml respectively, p<0.0001). IFN-γ production was higher in HIV-infected participants compared to HIV-uninfected controls (median IFN-γ 512 vs 223 IU/ml, p<0.001; Fig 1).

In a multivariate model using variables of HIV status, age and sex, being HIV infected was independently associated with a median increase of IFN- γ production of 239 IU/ml (p = 0.002). We also tested for potential interactions of age and sex within the control population but there was no evidence of this. There were no differences between HIV-infected participants on and off ART (p = 0.94).

### Clinical associations of IFN-γ production in HIV-infected participants

**Fig 2:**
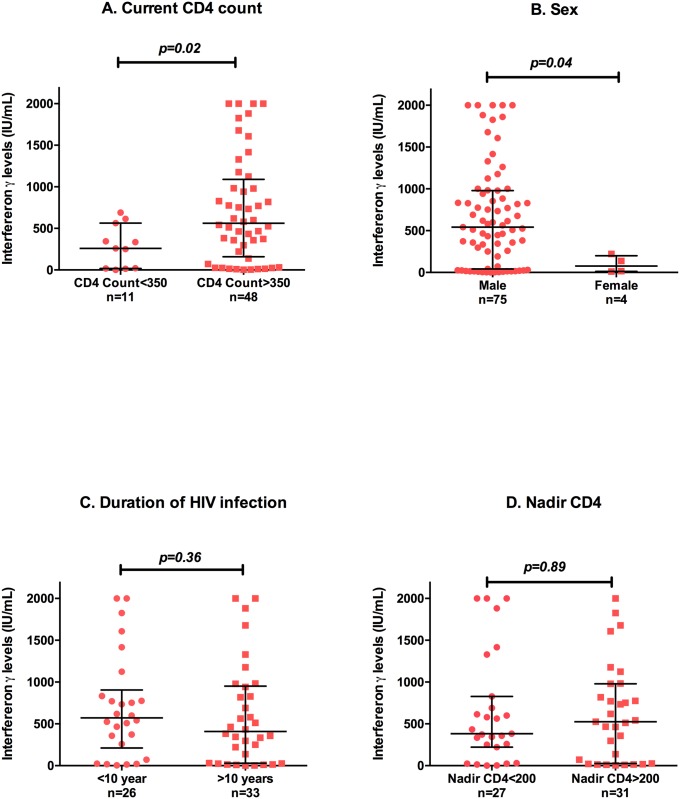
Innate and adaptive IFN-γ production ex vivo in HIV infected participants with horizontal error bars representing the median and interquartile range. A. CD4 count in treated participants, B. sex, C. duration of HIV infection and D. nadir CD4 count.

We then asked whether there was any association between IFN-γ production and any clinical parameters in the HIV-infected participants. IFN-γ production in HIV-infected participants on ART was significantly higher in participants with a current CD4+ T-cell count >350 cells/μl compared to a CD4+ T-cell count <350 cells/μl (median IFN-γ 561 vs 259 IU/ml p = 0.02; Fig 2). A statistically significant difference was also seen in male HIV-infected compared to female participants (median IFN-γ 542 vs 77 IU/ml p = 0.04 Fig 2), although the number of HIV-infected female participants was small (n = 4). No gender differences were observed in the male and female control population (median IFN-γ 241 vs 218 IU/ml p = 0.26). There were no significant associations between IFN-γ responses and age (p = 0.1), plasma HIV RNA (p = 0.56), nadir CD4+ T-cell count (p = 0.89), CD8+ T cell count (p = 0.18) or duration of known HIV infection (p = 0.36). Using a multivariable analysis with 6 variables, neither CD4 count nor sex were independently predictive of IFN production.

## Discussion

Using a high throughput novel assay assessing both adaptive and innate immunity, this study showed marked increases in IFN-γ production in HIV-infected patients compared to HIV-uninfected controls. The IFN-γ responses seen in HIV-infected participants was more than twice that of healthy controls, suggesting marked overall increased production of IFN-γ in the setting of HIV infection both on and off ART and with high CD4+ T-cells.

The high level of IFN-γ production in HIV infected participants on or off ART was in keeping with known immune dysfunction well described in HIV infection [2]. Even in a population of HIV-infected participants with high CD4+ T-cells counts, the overall magnitude of response remains markedly increased compared to healthy controls. However, it is unclear whether these high IFN-γ levels have deleterious or beneficial effect since in this study we did not assess clinical outcomes. The observation of higher IFN-γ levels with improved CD4+ T-cells differed to the results of previous studies which associated persistent immune dysfunction with poor recovery of CD4+ T-cells [2,9]. A potential explanation for this difference could be that the current assay examined stimulated responses to both CD3 and TLR7/8, whereas most studies of immune activation in HIV are performed using unstimulated cells, for example expression of HLA-DR, CD38 and other markers [10]. Plasmacytoid dendritic cells (pDCs) and NK cells may be important in TLR7 responses [11,12]. Changes in circulating (pDCs) dependent on CD4+ T-cells have been described in HIV [13] and since TLR7 is expressed on pDCs which are known potent producers of type I IFN, changes in pDC number and function may lead to changes in T cell activation and IFN levels [14,15]. The mechanism for NK and pDC control of IFN production induced by anti-CD3 is unclear but TLR7 agonists have also been shown to reduce Treg function resulting in increased IFN-γ in PBMCs [16]. Sex was also associated with differences in IFN-γ production, with female HIV infected participants showing a significantly reduced level of IFN-γ. Previous studies in HIV have also shown sex differences in the TLR7 innate pathway with increased IFN-α levels [17,18]. Gender differences in NK cell production of IFN-γ in a healthy population have been reported [19], however this was not observed in our study.

The only other study to evaluate the QuantiFERON Monitor^®^ immune function assay to date has been in a cohort of liver transplant recipients [8]. The assay was able to distinguish between participants with poor immune function in the early post-transplant period (median 31 days) from late post-transplant participants (median 6.2 years) as observed by a significantly lower mean IFN-γ level [8]. One of the most remarkable and significant differences between our study population and the one performed by Sood et al, was the overall magnitude of the IFN-γ response. Liver transplant recipients at the peak of their immunosuppressive period recorded mean IFN-γ levels of 3.8 IU/ml, compared to a median IFN-γ of 297 IU/ml in HIV-participants with CD4+ T-cells <350 cells/ μl. Samples were processed in the same laboratory from both studies so technical differences are unlikely. As the response to both adaptive and innate stimuli was combined in a single tube, we were unable to differentiate which stimulus induced the high production of IFN-γ observed in HIV infection. Overall these results demonstrate that even in chronic well suppressed HIV infection, there is marked increased and persistent IFN-γ production in response to these specific stimuli.

There were several limitations to this study. First, this was a cross sectional study and we were unable to confirm the exact duration of HIV infection. This may have confounded our comparisons between HIV-infected participants on and off ART. Secondly, the QFM only measured production of the cytokine IFN-γ, and not other cytokines which are likely important in immune activation [10,20]. Thirdly, the assay did not measure constitutive plasma and cell IFN-γ levels, but others have demonstrated IFN-γ levels are low in HIV infection [21,22].

This assay provides a simple standardised measurement of cell mediated immunity which can be performed in a basic laboratory without the need for specialised equipment; providing unique information on innate immunity in addition to markers of adaptive immunity. The assay however, does not simply quantify immune competence but rather also measures immune activation which is a key component of HIV pathogenesis. The study population and control population differed in their age and sex distribution, although in a multivariate analysis we found no relationship between IFN- γ production and either age or sex. Finally, longitudinal studies would be required to determine whether this assay predicts important clinical outcomes, either alone or in combination with quantification of other inflammatory cytokines [23].

In conclusion, this novel assay measuring IFN-γ production following stimuli of the adaptive and innate immune response, showed a marked increase in IFN-γ production in HIV-infected participants compared to HIV-uninfected controls, regardless of whether participants were on or off ART. Further studies are warranted to determine the cellular correlates for these changes and if these changes in QuantiFERON-Monitor^®^ are associated with clinical outcomes in HIV-infected individuals on ART.

## References

[1] French MA, King MS, Tschampa JM, da Silva BA, Landay AL (2009) Serum immune activation markers are persistently increased in patients with HIV infection after 6 years of antiretroviral therapy despite suppression of viral replication and reconstitution of CD4+ T cells. J Infect Dis 200: 1212–1215. 10.1086/605890 19728788

[2] Hunt PW, Martin JN, Sinclair E, Bredt B, Hagos E, Lampiris H, et al (2003) T cell activation is associated with lower CD4+ T cell gains in human immunodeficiency virus-infected patients with sustained viral suppression during antiretroviral therapy. J Infect Dis 187: 1534–1543. 10.1086/374786 12721933

[3] Jain V, Hartogensis W, Bacchetti P, Hunt PW, Hatano H, Sinclair E, et al (2013) Antiretroviral therapy initiated within 6 months of HIV infection is associated with lower T-cell activation and smaller HIV reservoir size. J Infect Dis 208: 1202–1211. 10.1093/infdis/jit311 23852127 PMC3778965

[4] Rajasuriar R, Khoury G, Kamarulzaman A, French MA, Cameron PU, Lewin SR (2013) Persistent immune activation in chronic HIV infection: do any interventions work? AIDS 27: 1199–1208. 23324661 10.1097/QAD.0b013e32835ecb8b PMC4285780

[5] Kuller LH, Tracy R, Belloso W, De Wit S, Drummond F, Lane HC, et al (2008) Inflammatory and coagulation biomarkers and mortality in patients with HIV infection. PLoS Med 5: e203 10.1371/journal.pmed.0050203 18942885 PMC2570418

[6] Tincati C, Bellistri GM, Casana M, Merlini E, Comi L, Bai F, et al (2009) CD8+ hyperactivation and senescence correlate with early carotid intima-media thickness in HIV+ patients with no cardiovascular disease. J Acquir Immune Defic Syndr 51: 642–644. 19628982 10.1097/QAI.0b013e3181add695

[7] Gazzola L, Bellistri GM, Tincati C, Ierardi V, Savoldi A, del Dole A, et al (2013) Association between peripheral T-Lymphocyte activation and impaired bone mineral density in HIV-infected patients. J Transl Med 11: 51 10.1186/1479-5876-11-51 23448662 PMC3598927

[8] Sood S, Cundall D, Yu L, Miyamasu M, Boyle JS, Ong SY, et al (2014) A novel biomarker of immune function and initial experience in a transplant population. Transplantation 97: e50–51. 24732902 10.1097/TP.0000000000000078

[9] Fernandez S, Price P, McKinnon EJ, Nolan RC, French MA (2006) Low CD4+ T-cell counts in HIV patients receiving effective antiretroviral therapy are associated with CD4+ T-cell activation and senescence but not with lower effector memory T-cell function. Clin Immunol 120: 163–170. 10.1016/j.clim.2006.04.570 16765088

[10] Lichtfuss GF, Hoy J, Rajasuriar R, Kramski M, Crowe SM, Lewin SR (2011) Biomarkers of immune dysfunction following combination antiretroviral therapy for HIV infection. Biomark Med 5: 171–186. 10.2217/bmm.11.15 21473720

[11] Jiang Y, Zhou F, Tian Y, Zhang Z, Kuang R, Liu J, et al (2013) Higher NK cell IFN-gamma production is associated with delayed HIV disease progression in LTNPs. J Clin Immunol 33: 1376–1385. 10.1007/s10875-013-9930-1 23996459

[12] Hart OM, Athie-Morales V, O'Connor GM, Gardiner CM (2005) TLR7/8-mediated activation of human NK cells results in accessory cell-dependent IFN-gamma production. J Immunol 175: 1636–1642. 16034103 10.4049/jimmunol.175.3.1636

[13] Soumelis V, Scott I, Gheyas F, Bouhour D, Cozon G, Cotte L, et al (2001) Depletion of circulating natural type 1 interferon-producing cells in HIV-infected AIDS patients. Blood 98: 906–912. 11493432 10.1182/blood.v98.4.906

[14] Kadowaki N, Ho S, Antonenko S, Malefyt RW, Kastelein RA, Bazan F, et al (2001) Subsets of human dendritic cell precursors express different toll-like receptors and respond to different microbial antigens. J Exp Med 194: 863–869. 11561001 10.1084/jem.194.6.863 PMC2195968

[15] Martinson JA, Roman-Gonzalez A, Tenorio AR, Montoya CJ, Gichinga CN, Rugeles MT, et al (2007) Dendritic cells from HIV-1 infected individuals are less responsive to toll-like receptor (TLR) ligands. Cell Immunol 250: 75–84. 10.1016/j.cellimm.2008.01.007 18334250 PMC2838175

[16] Voo KS, Bover L, Harline ML, Weng J, Sugimoto N, Liu YJ (2014) Targeting of TLRs inhibits CD4+ regulatory T cell function and activates lymphocytes in human peripheral blood mononuclear cells. J Immunol 193: 627–634. 10.4049/jimmunol.1203334 24928999 PMC4347808

[17] Meier A, Chang JJ, Chan ES, Pollard RB, Sidhu HK, Kulkarni S, et al (2009) Sex differences in the Toll-like receptor-mediated response of plasmacytoid dendritic cells to HIV-1. Nat Med 15: 955–959. 10.1038/nm.2004 19597505 PMC2821111

[18] Chang JJ, Woods M, Lindsay RJ, Doyle EH, Griesbeck M, Chan ES, et al (2013) Higher expression of several interferon-stimulated genes in HIV-1-infected females after adjusting for the level of viral replication. J Infect Dis 208: 830–838. 10.1093/infdis/jit262 23757341 PMC3733517

[19] Snyder-Cappione JE, Tincati C, Eccles-James IG, Cappione AJ, Ndhlovu LC, Koth LL, et al (2010) A comprehensive ex vivo functional analysis of human NKT cells reveals production of MIP1-alpha and MIP1-beta, a lack of IL-17, and a Th1-bias in males. PLoS One 5: e15412 10.1371/journal.pone.0015412 21082024 PMC2972714

[20] Roff SR, Noon-Song EN, Yamamoto JK (2014) The Significance of Interferon-gamma in HIV-1 Pathogenesis, Therapy, and Prophylaxis. Front Immunol 4: 498 10.3389/fimmu.2013.00498 24454311 PMC3888948

[21] Watanabe D, Uehira T, Yonemoto H, Bando H, Ogawa Y, Yajima K, et al (2010) Sustained high levels of serum interferon-gamma during HIV-1 infection: a specific trend different from other cytokines. Viral Immunol 23: 619–625. 10.1089/vim.2010.0065 21142447

[22] Mihret A, Abebe M, Bekele Y, Aseffa A, Walzl G, Howe R (2014) Impact of HIV co-infection on plasma level of cytokines and chemokines of pulmonary tuberculosis patients. BMC Infect Dis 14: 125 10.1186/1471-2334-14-125 24592945 PMC3974017

[23] Roberts L, Passmore JA, Williamson C, Little F, Bebell LM, Mlisana K, et al (2010) Plasma cytokine levels during acute HIV-1 infection predict HIV disease progression. AIDS 24: 819–831. 20224308 10.1097/QAD.0b013e3283367836 PMC3001189

